# Wellbore Stability in Oil and Gas Drilling with Chemical-Mechanical Coupling

**DOI:** 10.1155/2013/720271

**Published:** 2013-07-10

**Authors:** Chuanliang Yan, Jingen Deng, Baohua Yu

**Affiliations:** ^1^State Key Laboratory of Petroleum Resource and Prospecting, China University of Petroleum, Beijing 102249, China; ^2^Department of Petroleum Engineering, China University of Petroleum, Beijing 102249, China

## Abstract

Wellbore instability in oil and gas drilling is resulted from both mechanical and chemical factors. Hydration is produced in shale formation owing to the influence of the chemical property of drilling fluid. A new experimental method to measure diffusion coefficient of shale hydration is given, and the calculation method of experimental results is introduced. The diffusion coefficient of shale hydration is measured with the downhole temperature and pressure condition, then the penetration migrate law of drilling fluid filtrate around the wellbore is calculated. Furthermore, the changing rules of shale mechanical properties affected by hydration and water absorption are studied through experiments. The relationships between shale mechanical parameters and the water content are established. The wellbore stability model chemical-mechanical coupling is obtained based on the experimental results. Under the action of drilling fluid, hydration makes the shale formation softened and produced the swelling strain after drilling. This will lead to the collapse pressure increases after drilling. The study results provide a reference for studying hydration collapse period of shale.

## 1. Introduction

Maintaining wellbore stability is an important issue in oil and gas industry [1–10]. In the process of drilling, the economic losses caused by wellbore instability reaches more than one billion dollar every year [11], and the lost time is accounting for over 40% of all drilling related nonproductive time [12]. It is also reported that shale account for 75% of all formations drilled by the oil and gas industry, and 90% of wellbore stability problems occur in shale formations [13–18]. When a well is drilled, the formation around the wellbore must sustain the load that was previously taken by the removed formation. As a result, an increase in stress around the wellbore and stress concentration will be produced [19–23]. If the strength of the formation is not strong enough the wellbore will be failure [24–28]. Wellbore stability is not only a pure rock mechanical problem, but also the interaction of drilling fluid and shale is a more important influence factor [29–35]. There are various chemicals in the drilling fluid which physically and chemically interact with shale formations. One hand, these interactions will result in the production of swelling stress [36–43]. On the other hand, it alleviates the mechanical strength of the wellbore wall rock [44–46]. Furthermore, it results in wellbore instability.

When studying the wellbore stability in shale, chemical factor must be combined with mechanical factor. Before the 1990s, the combinations are mainly on experimental study. Chenevert studied mechanical properties of shale after hydration since 1970s [44]. The results showed that the hydration would decrease the shale strength. After 1990s, the combinations came into a quantitative research stage. Yew et al. (1990) [29] and Huang et al. (1995) [47] combined shale hydrated effect quantitatively into the mechanical model based on thermoelasticity theory. Their method attributed the rock mechanical properties change with total water content. Take shale as a semipermeable membrane, Hale et al. (1993) [48, 49], Deng et al. (2003) [50], and Zhang et al. (2009) [51] introduced equivalent pore pressure to study interaction of shale and water base drilling fluid. Ghassemi et al. (2009) [52] proposed a linear chemo-thermo-poroelasticity coupling model, which considers the influence of chemical potential and temperature. Wang et al. (2012) [53, 54] built a fluid-solid-chemistry coupling model, in which they considered electrochemical potential, fluid flow caused by ion diffusion.

The chemical effect of drilling fluid on shale can be ultimately attributed to the variation of rock mechanical properties and stress around the wellbore. Water migration in shale is the basement of all wellbore stability models with chemical-mechanical coupling. A new experimental equipment to measure in situ water diffusion coefficient of shale is developed in this paper. And a sample model to evaluate time-dependent collapse pressure with chemical-mechanical coupling is presented. 

## 2. Experimental Research on the Hydration of Shale

The free water and ion will penetrate into shale under the driving force of chemical potential and pressure difference between the pore fluid and drilling fluid [55–58]. Water content of shale changes by various mechanisms such as osmosis flow, viscous flow and capillary flow. Osmosis flow, driving force is due to chemicals and ions with different composition in drilling fluid and pore fluid. In order to evaluate the hydration of drilling fluid, the coefficient of water absorption and diffusion and the swelling ratio must be determined first [36].

### 2.1. Experimental Research on the Water Absorption of Shale

#### 2.1.1. Experimental Equipment

**Figure 1:**
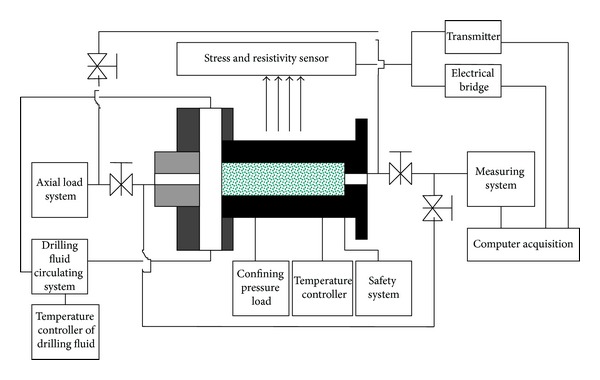
The experimental equipment sketch.

Cherevent let one end face of shale sample contact with drilling fluid and the other end face wrapped up by plastic film, then he measured the water content increment in different location. But his experiment can only be conducted in room temperature and with zero confining pressure. But during drilling process in deep formation, it is in the condition of high temperature and high pressure. Shale hydration is influenced by temperature and pressure seriously, so his experimental result was inconsistent with actual drilling. In order to test the coefficient of water diffusion of shale, we developed an in situ test equipment of water diffusion coefficient which can fit the downhole temperature and pressure condition while drilling (Figure 1).

Technical parameters of this designed equipment are as the following.

1. Temperature: room temperature to 150°C, which can imitate the temperature condition of the formation with 5000 meters depth.
2. Pressure: confining pressure 0 MPa to 70 MPa, axial pressure 0 MPa to 200 MPa.
3. Imitate the maximum differential pressure of drilling fluid with 10 MPa.
4. Sample size: *ϕ* 25 mm × 50 mm.

The experimental process are as follows.

1. Determine the original water content of the rock samples first, wrap the samples with separation sleeve, and put into the core holder. Put the drilling fluid into the tank and check the test system to make sure it is in good condition.
2. Turn on the temperature controller, warm the core samples to the same temperature with downhole condition. Then load the confining pressure and axial pressure to proper value and start timing.
3. During the test, data acquisition control system is used to keep the test values constant.
4. Cooling uninstall when the test time reaches the predetermined value (50 hours in this research), remove the rock samples quickly, and measure the water content at different distance from the end face.

#### 2.1.2. Coefficient of Water Diffusion

According to conservation of mass, water diffusion equations can be established. Supposing *q* is mass flow rate of the water diffusion, *C*_*S*_(*r*, *t*) is the weight percentage of water at the time *t* and distance *r* away from the well axis; according to conservation of mass requirement, the following equation can be presented:

(1) ∇q=∂CS∂t.

Consider that

(2) q=Deff∇CS,

where ∇ is the gradient operator and *D*_eff_ is the coefficient of water diffusion.

According to the above equations, water diffusion equation can be established as follows:

(3) ∂CS∂t−1r∂∂r(r∂CS∂r)Deff=0.

And the boundary conditions are

(4) t=0, rw≤r≤∞, CS=C0,t>0, r=rw, CS=Cdf,t>0, r→∞, CS=C0,

where *r*_*w*_ is the wellbore radius; *C*_*df*_ is the saturated water content of shale; *C*_0_ is the original water content.

$\text{Sign}\;u=r/\sqrt{D_{\text{eff}}t}$, *C*_*S*_(*r*, *t*) = *ϕ*(*u*), then the following equations can obtain that

(5) ∂CS∂t=dϕdu·∂u∂t=dϕdu(−12u)t−1,∂CS∂r=dϕdu·∂u∂r=dϕdu1Defft,∂2CS∂r2=ddr(dϕdu1Defft)=1Defft∂2ϕ∂u2

Insert (5) to (3), then (6) can be obtained:

(6) ϕ′′=−(u2+1u)ϕ′.

Equations (7) and (8) can obtain by integrating (6) that

(7) ϕ′=−Au−1e−u2/4,

(8) $$\begin{array}{c} C_{S}\left(r,t\right)=\phi \left(u\right)=\frac{A}{2}\int_{u^{2}/4}^{+\infty }x^{-1}e^{-x}dx+B. \end{array}$$

Combining (8) and (4) the following equations can obtain that.

(9) A=2(Cdf−C0)∫rw2/4Defft+∞x−1e−xdx,B=C0.

Thus, the water content of shale formation around the wellbore can be written as follows:

(10) CS =C0+(Cdf−C0)  ×[1+2π∫0∞e−Deff·ζ2·tJ0(ζr)N0(ζrw)−N0(ζr)J0(ζrw)J02(ζrw)+N02(ζrw)             ·dζζ],

where *J*_0_( ) and *N*_0_( ) are the zero order of Bessel's functions of group one and two, respectively.

In a short period of time after drilling and within a short distance from the wellbore wall, (10) can be simplified to

(11) CS=C0+(Cdf−C0)rwrerfc⁡(r−rw2Defft).

**Figure 2:**
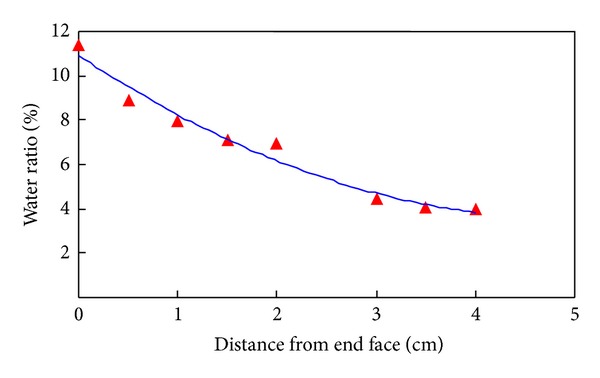
Experimental results of water diffusion in shale.

The water diffusion character of shale is measured using this designed experiment equipment. All the shale core samples used in this paper were collected from Bohai Bay Basin of China. The drilling fluid which contacted the shale in this experiment was KCL drilling fluid. The experimental confining pressure was 20 MPa, and the differential pressure of the fluid was 6 MPa. Core samples were taken out after 50 hours and then cut into pieces to measure the water content of each piece. Three samples were tested in this research. The experimental results of core sample 1-1 are shown in Figure 2. Substituting the experimental results into (11), the coefficient of water diffusion of shale can be obtained. All the calculated water diffusion coefficients and clay mineral contents of these three samples are shown in Table 1. Smectite is the mineral most prone to hydration [59, 60], and the water diffusion coefficient is higher with more smectite.

### 2.2. Chemical Effect of Drilling Fluid on Shale Mechanical Properties

**Figure 3:**
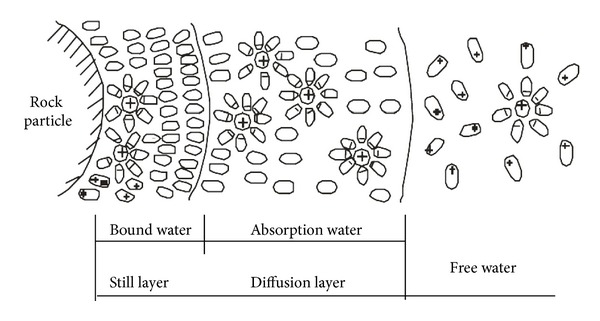
Water existing states in shale [63].

**Figure 4:**
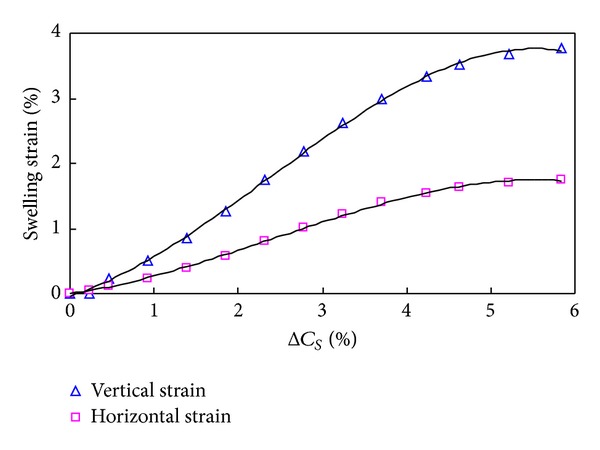
Experimental results of shale swelling.

The mechanical properties of shale can be altered seriously after contacting with drilling fluid. Existing forms of water in shale mainly include water vapor, solid water, bound water, adsorption water (film water), capillary water, and gravity water (free water) (Figure 3). Owing to the direct contact with drilling fluid around the wellbore, the free water of drilling fluid diffuses into shale under physical and chemical driving force. During the drilling process, the absorption water will increase, and the diffusion layer of rock particle will thicken, which will cause volume increase of shale and produce swelling stress. In order to calculate the swelling stress caused by hydration, the relation between water absorption and the swelling must be researched first through experiments. The experiment methods are similar to that used by Yew et al. [29]. The experimental results are shown in Figure 4.

The experiment results show that the swelling strain in the direction perpendicular to the deposition surface is larger than that of the parallel direction, which is resulted from the difference of drainage and stress conditions in different directions in sedimentation [61, 62]. The relationship of water content and swelling strain is as follows:

(12) εv=−324.8(ΔCS)3+23.5(ΔCS)2+0.37ΔCS,εh=−150.8(ΔCS)3+10.9(ΔCS)2+0.17ΔCS,

where *ε*_*v*_ and *ε*_*h*_ are the swelling strain in the direction perpendicular and parallel to the deposition surface, respectively; Δ*C*_*S*_ is the water content increment.

When contacting with drilling fluid, water sensitivity minerals of shale will absorb water and make a chemical reaction. Clay mineral in shale will react with the ions in drilling fluid [63, 64]:

(13) K0.9Al2.9Si3.1O10(OH)2+nH2O  →K0.9Al2.9Si3.1O10(OH)2nH2O.

The properties of shale particle will change by above chemical reaction, and then weaken the cohesive force between shale particles, which will soften the shale and weaken the strength. 

**Figure 5:**
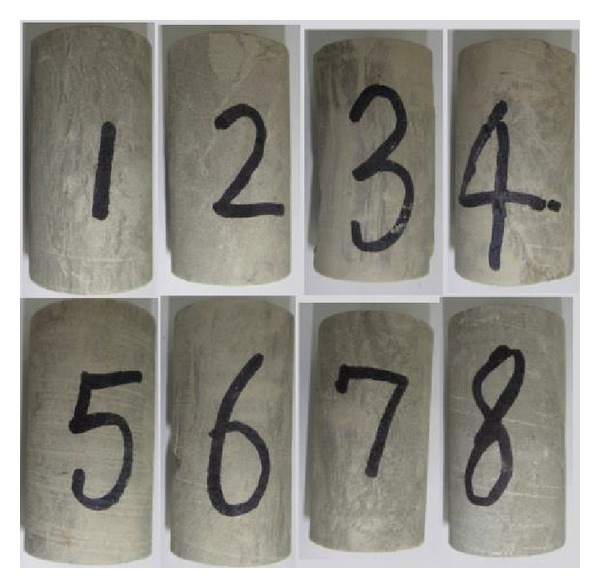
Standard samples of shale.

**Figure 6:**
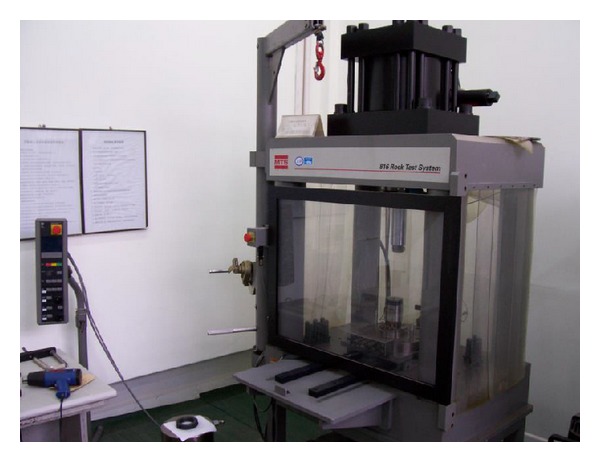
MTS-816 rock test system.

In order to evaluate wellbore stability after shale hydration, the mechanical properties of shale after hydration must be researched. In order to ensure the uniformity of the core samples used in the experiment, the compressive acoustic wave velocities of core samples were tested. Only the samples whose velocity is close are chosen. The core samples are shown in Figure 5. The samples are immersed in KCL drilling fluid at the temperature of 55°C. MTS-816 rock test system (Figure 6) was adopted to test shale mechanical properties. The experiment results are showed in Table 2.

The relationship of rock mechanical parameters with water content increment is obtained by the experimental results:

(14) E=5.5×103e−4.5ΔCS,v=0.26+2.1ΔCS,

(15) UCS=16.3−1.49ΔCS,

where *E* is the Young's modulus; *v* is the Poisson's ratio; UCS is the unconfined compressive strength.

Assume that the variation of shale cohesion with water content increment is the same with the variation of UCS:

(16) τ=τ′−1.49ΔCS,

where *τ* is the cohesion; *τ*′ is the initial cohesion before immersing, which is tested as 5.2 MPa.

The shear failure of wellbore obeys Mohr-Coulomb strength criterion. Mohr-Coulumb strength criterion can be expressed by principal stress [23]:

(17) σ1=σ3ctg2(45°−φ2)+2τ·ctg(45°−φ2),

where *σ*_1_ and *σ*_3_ are the maximum and minimum effective principal stresses, respectively; *φ* is the internal friction angle.

There is *σ*_3_ = 0 in the uniaxial compression experiment. Inserting (15) and (16) into (17), the variation of internal friction angle with water content increment can be obtained:

(18) φ=arctan(16.3−1.49ΔCS10.4−2.98ΔCS).

## 3. Wellbore Stability Model with Chemical-Mechanical Coupling 

Assuming that the shale is linear elastic material, the constitutive equation in plane strain is shown as follows:

(19) εr=[σr−v(σθ+σz)]E+εh,εθ=[σθ−v(σr+σz)]E+εh,εz=[σz−v(σr+σθ)]E+εv,

where *σ*_*r*_, *σ*_*θ*_, and *σ*_*z*_ are radial, tangential, and vertical stresses, respectively; *ε*_*r*_, *ε*_*θ*_, and *ε*_*z*_ are radial, tangential, and vertical straines, respectively.

The formation is replaced by fluid column pressure after drilling. The original stress balance around the wellbore is broken. A new balance will be built. The stress balance equation is as follows [65]:

(20) dσrdr+σr−σθr=0,

where *σ*_*ij*_ is the total stress tensor and *f*_*i*_ is the volume force.

The strain components and displacement components of the formation should meet the following geometric equation [65]:

(21) εr=dudr,εθ=ur,

where *ε*_*ij*_ is total strain tensor and *u*_*i*_ is the displacement component.

Inserting (19) and (20) to (21), the following obtains that.

(22) rd2σrdr2+(3−rE1dE1dr+2vrv2−1dvdr)dσrdr   +(4v+1v2−1dvdr−1E12v−1v−1dE1dr)σr  =E1(l+v)v2−1dεvdr+E1εvv2−1dvdr.

The boundary conditions for drilling are as follows [66]:

(23) σr=Pw, r=rw,σr=S, r=∞,

where *P*_*w*_ is under the fluid column pressure and *S* is the far field horizontal in situ stress.

Solving (22) and (23) using finite-difference method, stress distribution around the wellbore and its change rules with drilling time are obtained. Combined with Mohr-Coulumb failure criterion, the time-dependent collapse pressure can be obtained.

## 4. Time-Dependent Collapse Pressure

Based on the above model and experimental results, the variations of mechanical parameters of shale around the wellbore and time-dependent collapse pressure are analyzed. The calculation parameters are as follows: well depth *H* = 1800 m, the initial water content *C*_0_ = 4%, saturation water content *C*_*df*_ = 11.4%, the water diffusion coefficient *D*_eff_ = 0.0238 cm^2^/h, and the wellbore radius *r*_*w*_ = 10.8 cm; the other parameters are obtained by the experimental results.

**Figure 7:**
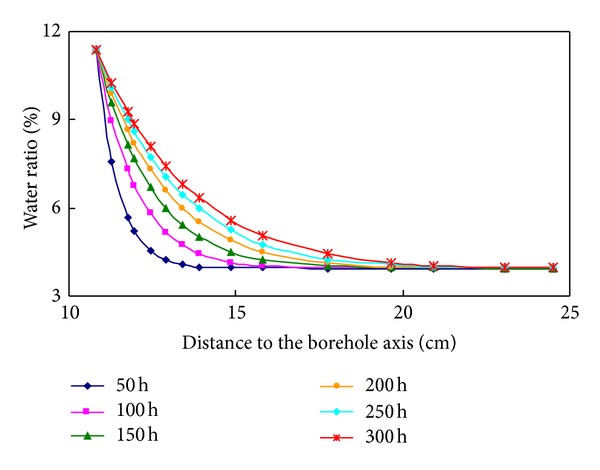
Water content distribution around the wellbore.

Figure 7 shows the variation of water content in shale formation around the wellbore with different open-hole time. The water content at the wellbore wall reaches to saturated state quickly after the wellbore is opened; in the same time, the water content would decrease with the increment of distance from the wellbore axis, and the decreasing rate is the highest near the wellbore wall. Thus, a hydrated area would develop around the wellbore. When the distance from the hole axis excesses 20 cm, the water content of shale almost no longer changes with the time increases, and the water content approaches the initial water content; in hydrated area, the longer the time, the more the shale water content when the distance is constant. 

**Figure 8:**
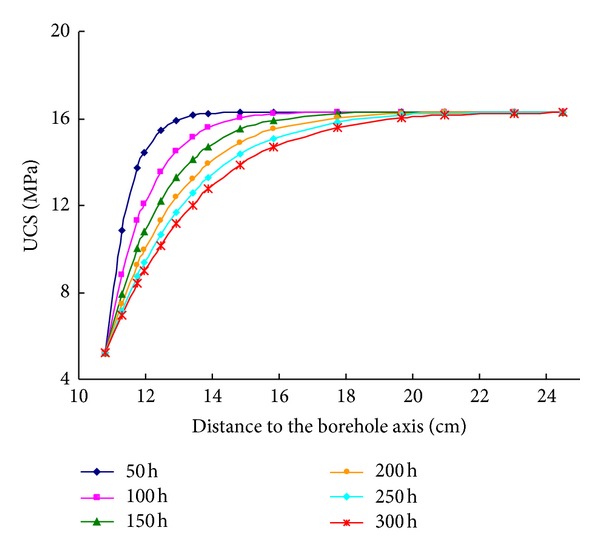
UCS distribution around the wellbore.

The distribution character of UCS of shale around the wellbore is presented in Figure 8. When a wellbore is opened, the UCS would decrease as the time increases. When the time is constant, the UCS would increase as the distance from the wellbore axis increases. The increasing rate near the wellbore wall is the highest. 

**Figure 9:**
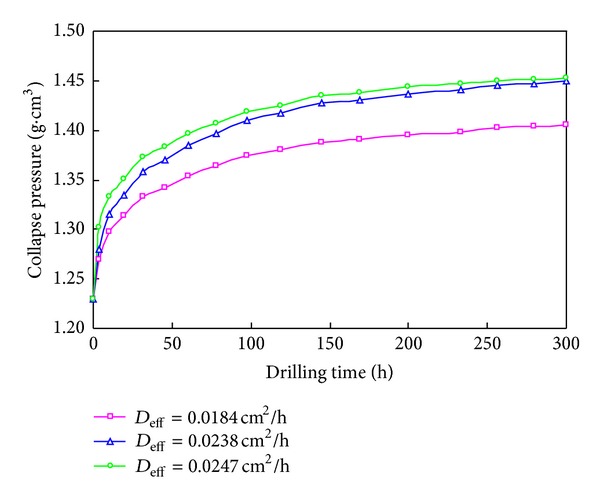
Time-dependent collapse pressure.

The variation of collapse pressure with drilling time under different water diffusion coefficients is shown in Figure 9. The results show that the collapse pressure increases rapidly in a short time after the wellbore opening due to shale hydration. Then the increasing rate of collapse pressure would decrease. At last, the collapse pressure would increase linearly with a very low increasing rate. After the wellbore is opened 10 days, the collapse pressure nearly no longer changes. According to Figure 9, after the wellbore opening, increasing the drilling fluid density to 1.45 g/cm^3^ gradually is more beneficial for long-time wellbore stability when the water diffusion coefficient *D*_eff_ = 0.0238 cm^2^/h. The higher the water diffusion coefficient is, the larger the increasing range of collapse is. On the other hand, the possibility of wellbore instability will be increasing with more smectite in shale. 

## 5. Conclusions

Water content at different distance from the end face of shale sample is measured using the designed equipment in the condition of downhole temperature and pressure. 

The water content of shale at the wellbore wall reaches to saturated state quickly when the wellbore is opened; the water content of shale would decrease with the increment of distance from wellbore axis, and the decreasing rate is the highest near the wellbore wall. 

Due to the impact of shale hydration, the strength of the circumferential formation around the well is gradually reduced with the increase of drilling time and increases with the increase of the distance away from the wellbore. 

Collapse pressure of shale increases sharply in a short time after drilling and then slows down. The collapse pressure is basically steady after several days of the open-hole time. The initial stable wellbore may collapse with the increase of the open-hole time.

Shale containing more smectite is more prone to react with drilling fluid. The possibility of wellbore instability of shale is higher with more smectite as the increasing range of collapse pressure is larger. 

## Figures and Tables

**Table 1:** Clay mineral contents and water diffusion coefficient of shale.

Core no.	Clay mineral total contents (%)	Clay mineral relative contents (%)				*D*_eff_
		Smectite	Illite	Kaolinite	Chlorite	(cm^2^/h)
1-1#	21.68	45	19	17	19	0.0238
1-2#	29.28	51	24	13	12	0.0247
1-3#	26.68	32	20	26	22	0.0184

**Table 2:** Experiment results of cores immersing in drilling fluid.

Immersing time (h)	0	2	6	12	24	48	96	192
Water content increment Δ*C*_*S*_ (%)	0	0.82	2.03	2.70	3.28	4.46	5.11	5.68
UCS (MPa)	16.30	13.56	11.76	12.24	9.48	8.28	8.52	6.60
Poisson's ratio	0.26	0.30	0.32	0.30	0.34	0.38	0.41	0.40
Elastic modulus (MPa)	5518.37	4055.17	2881.29	2176.90	2111.45	1759.96	2165.25	1599.73
